# Risk Stratification for Thyroid Malignancies in Chronic Lymphocytic Thyroiditis

**DOI:** 10.3390/cancers17121964

**Published:** 2025-06-12

**Authors:** Anna Krzentowska, Aleksander Konturek, Filip Gołkowski, Anna Merklinger-Gruchała, Marcin Barczyński

**Affiliations:** 1 Department of Endocrinology and Internal Medicine, Medical College, Andrzej Frycz Modrzewski Krakow University, 30-705 Kraków, Poland; fgolkowski@uafm.edu.pl; 2 Department of Endocrine Surgery, Faculty of Medicine, Jagiellonian University Medical College, 31-501 Kraków, Poland; aleksander.konturek@uj.edu.pl (A.K.); marcin.barczynski@uj.edu.pl (M.B.); 3 Faculty of Health Sciences, Medical College, Andrzej Frycz Modrzewski Krakow University, 30-705 Kraków, Poland; amerklinger-gruchala@uafm.edu.pl

**Keywords:** chronic lymphocytic thyroiditis, thyroid malignancy, prognosis

## Abstract

**Background**: Our study investigated the association between chronic lymphocytic thyroiditis (CLT) and thyroid cancer (TC). **Methods**: A retrospective review of 1670 patients who underwent thyroid surgery between October 2022 to February 2025 was performed. The clinicopathological characteristics of patients with TC and CLT were collected. CLT was diagnosed histopathologically. **Results**: Patients with a positive CLT result (60.39%) compared to a negative CLT result (34.12%) had more frequently TC (*p* < 0.001), predominantly PTC. In univariate analysis, patients with malignancy + CLT-positive compared to malignancy + CLT-negative were found to be younger in age (*p* < 0.001), female gender (*p* < 0.001), smaller tumours focus (*p* = 0. 013), smaller mass of thyroid removed (*p* < 0.001) and more often Bethesda category V and VI (*p* = 0.019), true positive fine needle aspiration biopsy (FNAB) for PTC (*p* = 0.009), and microcarcinoma (*p* = 0.021). There were no differences in multifocality, the presence of LNM, or the number of LNMs. The location of metastases at neck lymph nodes was at the borderline of significance (*p* = 0.065). In multivariate analysis, after accounting for Bethesda and age, CLT+ was found to increase the risk of TC by 73% (OR = 1.73; 95%CI, 1.15-2.29), while the risk of PTC increased more than 2-fold (OR = 2.12; 95% CI, 1.45–3.11). CLT had no statistically significant effect on the presence of LNM. **Conclusions**: We found that CLT is a risk factor for TC. One should be vigilant concerning the coexistence of these two diseases. We suggest that total thyroidectomy should be considered in patients referred for thyroid nodules and suspected CLT. However, this issue requires further research.

## Simple Summary

We retrospectively assessed the association between the presence of chronic lymphocytic thyroiditis (CLT) and thyroid cancer (TC). We analysed one thousand six hundred and seventy patients referred for surgery at the Department of Endocrine Surgery at the University Hospital in Kraków. A group of five hundred and eighty-eight patients with confirmed malignant thyroid tumours was identified. CLT was confirmed histopathologically. Clinicopathological differences between the Malignancy CLT-positive and Malignancy CLT-negative groups were analysed. CLT was found to have a statistically significant effect on the presence of TC. The most common type of TC was papillary thyroid cancer (PTC). CLT did not affect the presence or location of neck lymph node metastases (LNMs).

## 1. Introduction

Chronic lymphocytic thyroiditis (CLT) known as Hashimoto thyroiditis (HT) is the most common type of autoimmune thyroid disease [1], with its incidence rising in recent years. The prevalence of the disease depends on the geographical region. The prevalence in Africa was 14.2%, Oceania 11.0%, South America and Europe 8.0%, North America 7.8%, and Asia 5.8% [2]. HT is a common cause of primary hypothyroidism. HT is biochemically confirmed using elevated values of thyroid-stimulating hormone (TSH) and the presence of thyroid peroxidase antibody (TPOAb) and/or thyroglobulin antibody (TgAb). CLT is a histopathological diagnosis of HT, but its effect on the occurrence of TC remains unclear. Many studies have addressed the coexistence of TC and HT [3,4,5,6,7], and attempts have been made to elucidate the mechanisms affecting the association between the presence of CLT and TC [8]. Papillary thyroid carcinoma (PTC) is the most frequently found thyroid malignancy [3,9,10,11,12], while follicular thyroid carcinoma (FTC) and other types of thyroid tumours, such as lymphoma, have been found less frequently [13]. A meta-analysis of 50 studies addressing the issue of TC in HT showed that CLT played a significant role in the development of PTC, medullary thyroid carcinoma (MTC), and lymphoma but not anaplastic carcinoma (ATC) or FTC [12]. Although PTC has the best prognosis, lymph node metastasis remains a concern and occurs in approximately 40-90% of patients [14,15]. Several studies have evaluated the risk of neck lymph node metastasis (LNM) in patients with TC and CLT [10,16,17,18,19]. It has been reported that patients with PTC+CLT have a better prognosis [19,20,21] and central lymph node metastases (CLNM) and extrathyroidal metastases are less frequently present [22]. Therefore, it has been hypothesised that lymphocytic infiltration represents a form of immune response that controls tumour growth and proliferation [7]. Indeed, one study confirmed that it is more common for PTC to spread to the lymph nodes of compartment VI in patients with HT than in patients without HT [10,23].

Numerous attempts have been made to determine the clinical and pathological differences in patients with PTC with or without CLT [23,24], showing that PTC with CLT is more common in women [4,24] and at a younger age [4,24,25]. However, some studies have indicated that PTC is more common in men [25]. Another factor is tumour size and capsule invasion frequency, in that patients with CLT and PTC more often have small tumours and less thyroid capsule invasion [24,25], although there are studies where the presence of CLT did not affect these parameters [23]. Another factor that has been analysed is multifocality [4,23,26,27,28]. Some studies confirm a higher risk of cancer multifocality in patients with PTC and CLT [4,9], while others indicate lower multifocality [23,26]. Dong et al. found that TPOAb > 1300IU/mL indicates multifocal PTC in patients with CLT [28]. A study by Paparodis et al. confirmed that incidentally detected microcarcinomas are more common in patients with CLT than in those with multinodular goitre (NG) or Graves’ disease (GBD) [29]. Studies have also investigated whether oxidative stress and inflammatory markers influence the coexistence of CLT and PTC, finding that these factors are higher in PTC than in PTC+CLT groups [30]. The Bethesda categories have been analysed for their significance in determining the malignancy of thyroid tumours in the presence of CLT, showing that the malignancy rate of thyroid nodules with atypia of undetermined significance (AUS)/follicular lesion of undetermined significance (FLUS) cytology is comparable regardless of CLT [31]. However, Vaghaiwalla et al. found a high predictive value for FNAB in patients with CLT and PTC [32]. The impact of the BRAFV600E mutation has also been analysed, showing that this mutation occurs less frequently when PTC coexists with CLT, as CLT and the BRAFV600E mutation act independently in the development and progression of thyroid cancer [33]. Another study on this mutation showed that patients with PTC-HT had significantly fewer BRAF mutations than patients with PTC (OR = 0.45) [34]. According to these authors, patients with BRAF (+) PTC-HT positive are more likely to have multifocal lesions (OR = 1.22) but less likely to have LNM (OR = 0.65) and extrathyroidal extension (OR = 0.55) than patients with BRAF (+) PTC. In addition, patients with BRAF (+) PTC-HT positive were more likely to have multifocal lesions (OR = 0.71), lymph node metastases (OR = 0.59), and extrathyroidal extension (OR (95% CI) = 0.72 (0.56-0.92), *p* = 0.01) than patients with BRAF (−) PTC-HT.

Given the numerous studies devoted to the issue of CLT and TC coexistence, we attempted a retrospective assessment of this problem in a large cohort of patients undergoing thyroid surgery at our high-volume endocrine surgery centre.

Objectives:

1. Primary Objective: To retrospectively evaluate the association between CLT and thyroid tumour malignancy, acknowledging the study’s constraints in establishing causality.
2. Secondary Objective: To identify potential risk factors (age, sex, tumour size, single/multifocal presentation, Bethesda category in FNAB) for thyroid tumour malignancy in patients with CLT, while recognizing the limitations of retrospective analysis.
3. Tertiary Objective: To assess the prevalence and anatomical distribution of lymph node metastases (LNM) in patients with coexisting CLT and thyroid cancer, within the confines of the study design.

## 2. Materials and Methods

### 2.1. Study Group

**Figure 1:**
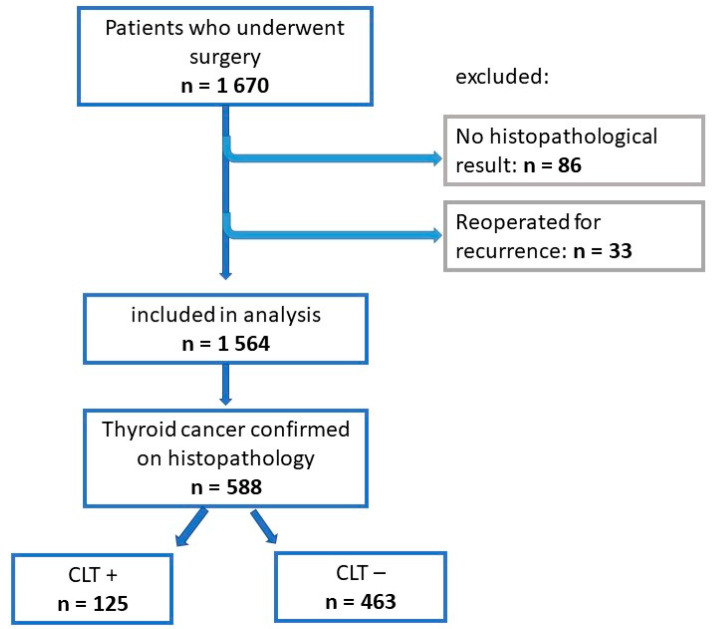
Flowchart of the inclusion or exclusion procedure.

We conducted a retrospective data analysis of 1670 patients who underwent surgery at the Department of Endocrine Surgery of the Jagiellonian University Medical College at the University Hospital in Krakow between October 2022 and February 2025. Patients were referred for surgery due to nodular goitre (SN), confirmation or suspicion of TC in FNAB, and hyperthyroidism in the course of Graves’ disease (GBD) or toxic multinodular goitre (TNG). Patients who did not obtain a histopathological result (86 cases) and who were referred again for revisional surgery due to local or nodal recurrence (33 cases) were excluded from the study. Ultimately, 1,564 patients were analysed. From this group, patients diagnosed with thyroid cancer (n = 588) with (n = 125) or without (n = 463) CLT in postoperative histopathological examination (HP) were identified. Patient data were anonymised and collected in the international EUROCRINE database [35]. This study was approved by the Bioethics Committee of the Andrzej Frycz Modrzewski University in Krakow (approval no. KB/UAFM/8/O/2025 of 23 January 2025). A visual representation of this selection process is provided in Figure 1.

### 2.2. Diagnosis and Evaluation

All patients underwent thyroid ultrasound (US) examination prior to surgery, and most underwent FNAB for suspicious thyroid nodules. Patients were referred for surgery from various medical clinics; therefore, not all patients underwent tumour assessment according to the EUTIRADS classification. Due to the lack of this data, they were not included in the analysis. FNAB cytological results were evaluated according to the Bethesda classification. Patients underwent total thyroidectomy or thyroid lobectomy. The extent of lymphadenectomy depended on the preoperative FNAB result. Central lymph node clearance was performed for Bethesda categories III, IV, V, and VI. In some patients with suspected/confirmed lateral node(s) metastasis, modified lateral neck dissection was performed. The postoperative material was evaluated in a histopathological (HP) exam, which was the main tool for confirming or ruling out the presence of CLT. TSH, TPOAb, and TGAb were not included in the analysis because the data were incomplete. This was because the patients were referred for surgery due to suspected or confirmed malignancy, hyperthyroidism in the course of GBD or TNG, or symptoms of compression. They were or were not treated with L-thyroxine. The TSH concentrations in patients treated with LT4 were the result of this treatment; we did not have TSH values prior to treatment initiation. Regarding TPOAb and TGAb concentrations, due to the nature of the diseases that were the indication for surgery, these tests are not mandatory; therefore, we did not have these data. Only a few patients had antibody results. Thyroid tumours were histopathologically evaluated according to the current WHO classification of thyroid tumours from 2022 [36]. If a malignant tumour was found, it was staged according to the American Joint Committee on Cancer and Union for International Cancer Control (AJCC/UICC) 2017, 8th edition tumour, node, and metastasis (TNM) system. Patients were divided into two groups depending on the presence or absence of CLT confirmed in HP. In both groups, age, sex, FNAB result, HP result, type of malignant thyroid tumour, type of thyroid, and lymph node surgery were assessed. Patients with diagnosed TC were then divided into two groups: Malignancy CLT-positive and Malignancy CLT-negative. The two groups were compared to assess whether there were differences in age, sex, tumour type and size, number of tumour foci, number of metastatic lymph nodes, LNM location, or thyroid mass removed. A multivariate analysis was then performed to determine the impact of CLT on the presence of TC and the extent of LNM.

### 2.3. Statistical Analysis

Continuous variables are expressed as mean  ±  SD or medians and quartiles (Q1, Q3), and were compared using an unpaired Student’s *t*-test or Mann–Whitney U test. The categorical variables are expressed as numbers (percentages). We used the χ^2^ test with a post hoc test and calculated standardised residuals or Fisher’s exact test to compare groups. Simple and multivariate logistic regression analyses were performed to determine whether CLT affects the risk of malignancy after standardisation to other predictors, such as gender, age, and Bethesda categories. Based on multivariate logistic regression analysis results, a nomogram was used to predict the risk of malignancy. The areas under curves (AUCs) of receiver operating characteristics (ROCs) were used to evaluate the prediction of malignancy risk. The Hosmer–Lemeshow test and McFadden coefficient were used as measures of goodness of fit for logistic regression models. Sensitivity, specificity, positive predictive value (PPV), and negative predictive value (NPV) were calculated. To assess the odds of having central or lateral metastases compared with having no metastases, multivariate multinomial logistic regression analysis was performed based on several predictors. To assess the quality of the model fit, the LR test, the Akaike Information Criterion (AIC), and McFadden’s R^2^ were used. In all multivariate analyses, age was included as a categorical variable; a cut-off value of 50 years was determined based on ROC analysis, where age was treated as a continuous predictor. This threshold provided the best balance between sensitivity and specificity, as indicated by the highest Youden’s J statistic. Results with *p*-values  <  0.05 were considered statistically significant. All statistical analyses were performed using Statistica 13 (StatSoft Inc., Tulsa, OK, USA) and jamovi version 2.6.26.

## 3. Results

### 3.1. Univariate Analysis of the Entire Study Group (n = 1564)

Among the 1564 patients enrolled, there were 276 men (17.6%) and 1288 women (82.4%). The reasons for referring a patient for the procedure were as follows: excluding malignant neoplasm, n = 523 (33.4%), malignancy, n = 298 (19.0%), compression symptom, n = 527 (33.7%), thyrotoxicosis, n = 98 (6.2%), and other n = 118 (7.5%). Based on a univariate analysis, statistically significant differences were found between the CLT-positive and CLT-negative groups in terms of age (*p* < 0.001), gender (*p* < 0.001), Bethesda category (*p* < 0.001), and HP results (*p* < 0.001). The CLT-positive group included more younger patients, more women, more cases classified as Bethesda categories V and VI, and more malignant lesions in HP. The demographic and pathological characteristics of the entire group were divided into CLT-positive and CTL-negative subgroups, as shown in Table 1.

### 3.2. Univariate Analysis of Patients with Malignancy CLT-Positive Compared to Malignancy CLT-Negative

A subgroup of patients with diagnosed TC (n = 588) was identified and divided into two subgroups, i.e., CLT-positive (n = 125) and CLT-negative (n = 463). Based on univariate analysis, statistically significant differences were found in the Malignancy CLT-positive group compared with the Malignancy CLT-negative group in terms of age (*p* < 0.001), gender (*p* < 0.001), tumour size (*p* = 0.013), Bethesda category (*p* = 0.019), positive FNAB for PTC (*p* = 0.009), the presence of microcarcinoma (*p* = 0.021), and thyroid weight removed (*p* < 0.001). PTC was the most common type of TC (*p* = 0.003). No statistically significant differences were found in the presence of LNM (*p* = 0.520), number of metastatic lymph nodes (*p* = 0.859) or T-TNM features (*p* = 0.155). The location of metastatic lymph nodes was borderline significant (*p* = 0.065). The CLT groups differed in their distribution across the Bethesda categories, which was confirmed using post-hoc analyses: in the CLT- positive vs. CLT- negative groups, Bethesda category II was less frequent (std. resid. = −2.25), while Bethesda category V was more frequent (std. resid. = 2.79). The results of the univariate analysis are presented in Table 2.

### 3.3. Multivariate Analysis of the Impact of CLT on the Risk of TC

To assess the impact of CLT on the occurrence of TC, a multivariate logistic regression was performed. CLT, Bethesda category, and age were included as predictors in the multivariate model. Gender was not included due to a lack of significant association with the dependent variable in the univariate model. EUTIRADS was not included due to numerous data deficiencies. After considering additional factors such as Bethesda and age, we found that increases the risk of malignant neoplasm by 73% (OR = 1.73; 95% CI 1.15–2.29). The results are presented in Table 3.

**Figure 2:**
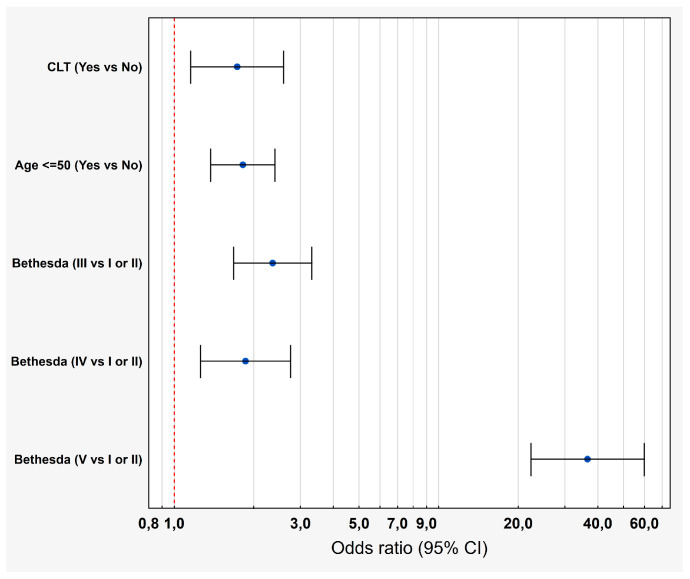
Multivariate analysis—assessment of TC probability in the case of coexisting CLT. Blue dots represent odds ratios with 95% confidence intervals (CI), shown on a probability scale. Varying CI widths result from the non-linear relationship between odds and probability.

In order to better visualize the above data, we present them in Figure 2.

**Figure 3:**
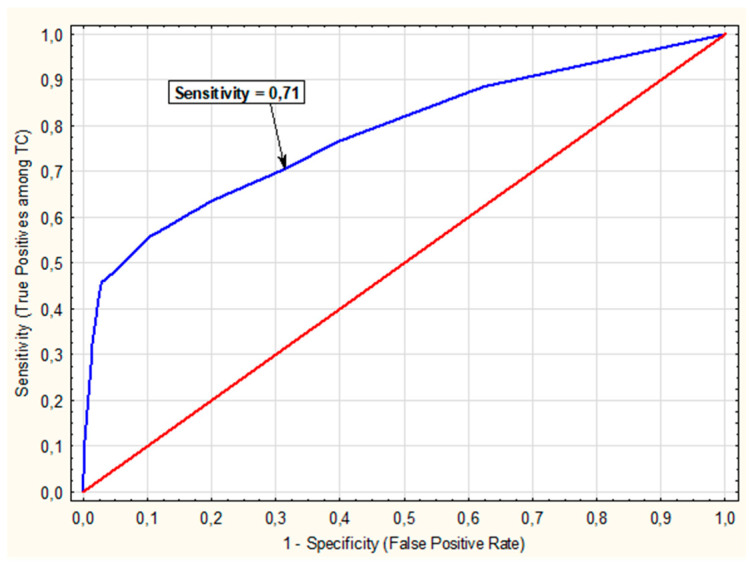
ROC curve showing the relationship between sensitivity and 1-specificity as a function of TC probability thresholds, indicating the cut-off point for which the model’s predictive values were calculated.

A ROC curve was plotted showing sensitivity and 1-specificity values depending on the probability of TC occurrence—Figure 3. The model’s high AUC value under a ROC curve of 0.78 (SE = 0.02) indicated that it could correctly classify patients (predict the risk of TC) in approximately 78% of cases. To effectively predict TC, based on the sensitivity and specificity graph, an appropriate decision threshold was selected in the logistic regression model, which was set at 0.25, the intersection point of the sensitivity and specificity lines. For the proposed decision threshold on the ROC curve, the following predictive measures were calculated: sensitivity = 70.55%, specificity = 68.61%, PPV = −51.92%, NPV = 82.90%. This model excluded the malignancy well (high NPV and sensitivity) but had only a moderate ability to confirm the malignancy (lower PPV and specificity). The model detected approximately 71% of TC cases (sensitivity), i.e., it effectively detected malignant tumours, which is important for minimising missed cases of this cancer (i.e., minimising false negatives). The relatively high NPV (82.90%) means that the probability of a patient classified as negative by the model not having malignancy (TC) is close to 83%, which means that the model is quite effective at ruling out the presence of the disease—only about 17% of people with a negative result may actually have TC. On the other hand, the relatively low PPV value (51.92%) indicated a 52% probability of the actual presence of TC in a patient being classified by the model as positive.

**Figure 4:**
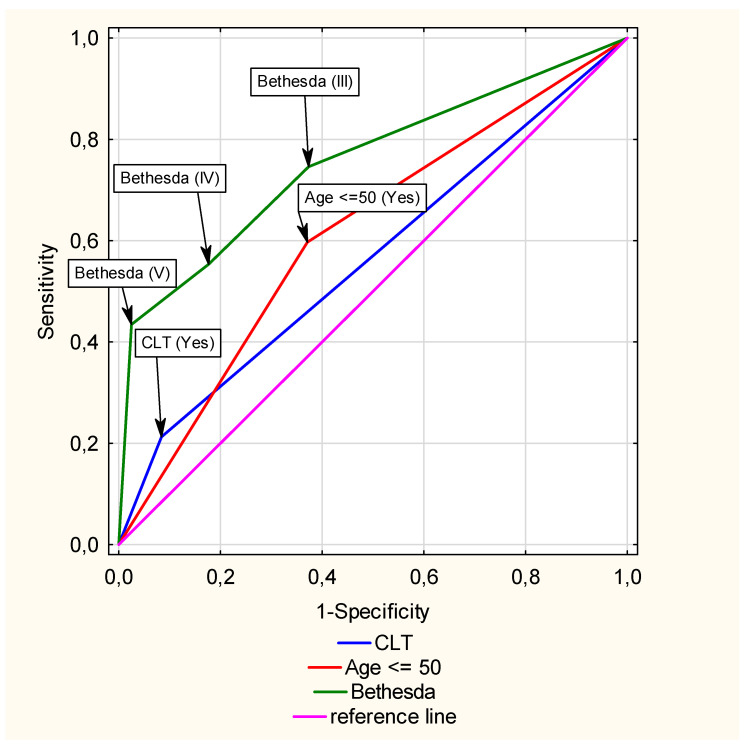
Diagnostic ability of selected predictors of thyroid cancer—comparison of ROC curves.

Additionally, we analysed the significance of differences in AUC values under ROC curves for CLT vs. Age and CLT vs. Bethesda (Figure 4), showing that the AUC for CLT = 0.56 (95% CI 0.53–0.59) significantly differed from the AUCs for Age < 50 years = 0.61 (95% CI 0.58–0.64) (*p* < 0.001) and Bethesda = 0.76 (95% CI 0.73–0.79), (*p* < 0.01).

This analysis indicated that the strongest single predictor of TC risk in the evaluated model was the Bethesda category, which had the highest AUC value (0.76), followed by age < 50 years (AUC = 0.61) and the presence of CLT (AUC = 0.56). CLT itself did not have a strong predictive value, but its AUC confidence interval did not include the value 0.5, indicating that knowledge of CLT status significantly improves the classification of malignant vs. benign tumours.

**Figure 5:**
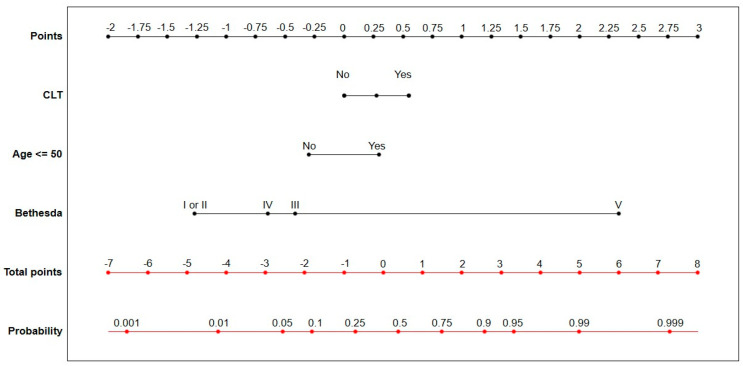
A nomogram predicting the risk of TC in patients with and without CLT based on age and Bethesda categories.

To visualise the predictive model and determine the individual probability of TC occurrence based on the values of the above predictive variables, a NOMOGRAM was created (Figure 5). Each parameter (CLT, age < 50. and Bethesda category) was assigned a score on a ‘points’ scale. The individual scores were then summed to generate a total score, which corresponded to a specific cancer risk indicated on the “probability” scale. To demonstrate how the nomogram was interpreted, the following example was considered: the simultaneous presence of CLT (0.5 points), age ≤ 50 years (0.30 points), and Bethesda category III (−0.5 points) yielded a total score of 0.30 points. This corresponded to an approximate 50% risk of cancer on the ‘probability’ scale.

### 3.4. Multivariate Analysis of the Impact of CLT on the Presence and Location of Lymph Node Metastases (LNM)

A multivariate analysis was performed on the patients with malignancy (n = 588), in order to assess whether CLT had an impact on the presence of LNM. Due to a substantial amount of missing data for the EUTIRADS variable (n = 320), it was excluded from the analysis. In the multivariate analysis, CLT did not affect the occurrence of LNM in patients with TC. CLT-positive patients had the same chance of developing metastases as patients without CLT (adjusted OR = 1.09; 95% CI 0.62–1.92). However, other parameters were found to influence the presence of LNM. Patients with multifocality had 25% higher odds of developing LNM than patients without multifocality, but the result was not statistically significant OR = 1.25 (95% CI 0.74–2.13). Younger patients (age ≤ 50 years vs. >50 years) had a 56% higher risk of LNM after adjusting for additional factors, but this association was not statistically significant (adjusted OR = 1.56; 95% CI 0.95–2.56). Both before and after adjusting for additional factors, the following variables had a significant impact on the occurrence of LNM: gender (adjusted OR = 0.45; 95% CI 0.26–0.77), i.e., women had a 55% lower chance of developing metastases than men) and the presence of microcarcinoma: (adjusted OR = 0.26; 95% CI 0.16–0.43), i.e., patients with microcarcinoma had a 74% lower chance of developing LNM than the group without microcarcinoma (Table 4).

Next, we analysed whether CLT impacts on the location of LNM in a group of patients with malignancy (n = 588). A multivariate multinomial logistic regression model was performed to compare the probability of central lymph node metastases (CLNM = pN1a) and lateral lymph node metastases (LLNM = pN1b) with no lymph node involvement (pNo). The model evaluated the odds of having central or lateral metastases compared to having no metastases, based on several predictors (Table 5).

In this model, CLT and multifocality were not significant predictors of CLNM (*p* = 0.52 and *p* = 0.43, respectively) or LLNM (*p* = 0.40 and *p* = 0.67, respectively). However, the presence of microcarcinoma consistently and significantly reduced the likelihood of metastasis in both central and lateral locations (by 64% and 97%, respectively). Younger patients were 1.75 times more likely to develop CLNM (OR = 1.75; 95% CI 1.01–3.03), but not LLNM (OR = 1.06; 95% CI 0.43–2.61). In females vs. males, there was a 77% lower likelihood of LLNM (OR = 0.23; 95% CI 0.10–0.58), but not CLNM (association at the borderline of statistical significance, *p* = 0.08).

The model showed a good fit with a significant LR test (χ^2^ = 66.8, *p* < 0.001). The AIC = 567.7 value suggested that the model was well-balanced in terms of complexity and fit, and a McFadden’s R^2^ of 0.11 indicated that it explained about 10.94% of the outcome variance.

### 3.5. Multivariate Analysis of the Impact of CTL on PTC Risk

Due to the frequent occurrence of PTC in the studied group (n = 495), multifactorial analyses were performed to assess the risk of this TC in CTL using simple and multivariate logistic regression. CLT, Bethesda category, and age were included as predictors in the multifactorial model. Gender was not included in the multivariate analysis due to a lack of a significant relationship with the dependent variable in the single-factor model. EUTIRADS was not included due to numerous data deficiencies, which would have radically reduced the sample size. The results are presented in Table 6.

## 4. Discussion

While many studies have attempted to examine and describe the relationship between CLT and TC, this connection remains controversial and there is still much uncertainty and conflicting results. This phenomenon may be due to the results of different studies varying depending on how HT is defined (from a clinical, biochemical, or histopathological point of view). HT is not in itself an indication for surgery, but CLT is often found as an additional diagnosis in patients referred for surgery due to thyroid tumours. The literature confirms that HT influences the presence of TC, mainly PTC [37,38]. The association between PTC and HT was first demonstrated in 1955 [39]. Many attempts have been made to elucidate the mechanisms responsible for the interrelationship between HT and TC [8,40]. On the one hand, it has been confirmed that HT increases the likelihood of PTC, and on the other, it improves its prognosis if PTC is confirmed. It has been suggested that the mechanism responsible for this cancer course is the regulation of gene expression, participation in common signalling pathways, and the formation of a specific tumour immune microenvironment (TIME) [41]. Recently, the roles of the TIME and molecular mechanisms in the development of thyroid cancer have been highlighted. In addition, the roles of inflammation and the immune system in the development of thyroid cancer have been investigated. Lymphocyte infiltration of thyroid tissue is present in HT and, although PTC is classified as a type of cancer and HT is classified as an autoimmune disease, there are overlapping genetic factors associated with both diseases [42]. In terms of gene expression, changes in PTC-HT are more pronounced than in HT alone, indicating a possible link between HT and PTC progression [42,43]. HT plays a significant role in the mechanisms of immune evasion by PTC, which is also associated with its influence on the TIME. In PTC-HT, compared with PTC alone, CD3+, CD4+, CD8+, B lymphocytes, and plasma cell levels are increased in thyroid tissue. The impact of HT on TIME in PTC was also analysed, demonstrating that HT affects PTC signalling by increasing the number of CD8+ cells. LNM is associated with an increase in M2 macrophages (CD163+) and vascular endothelial growth factor (VEGF) expression in PTC, while HT affects LNM through various mechanisms [44]. Recent large cohort studies and meta-analyses have clarified the prognostic impact of coexisting HT in PTC. For example, Yang et al. showed that DTC co-presenting with HT is associated with a low risk of advanced DTC and presents a low risk for all-cause and DTC-related death. They demonstrated that PTC-HT positive had significantly smaller tumours and less aggressive disease; HT was associated with markedly lower all-cause mortality (HR ≈0.71) and disease-specific mortality (HR ≈0.33) [45]. Xue et al. reviewed the literature to analyse the immunological and molecular mechanisms underlying the interaction between HT and PTC [46]. They found that PTC-HT positive exhibited characteristic features of the immune microenvironment, such as the role of regulatory T cells (Tregs), IFN-γ-mediated activation of the CXCR3A-CXCL10 signalling axis and activation of the NF-κB pathway. The role of TSH, RET/PTC gene rearrangements, and changes in STAT6 and DMBT1 gene expression levels in PTC development were also highlighted. In terms of molecular alterations, HT-associated PTC has distinctive features. A systematic review showed that BRAF^V600E mutations are significantly less frequent in PTC with HT (OR ≈0.45) than in conventional PTC, which may underlie the less aggressive phenotype and explain the slow clinicopathological course of PTC-HT positive [34]. At the same time, the immune infiltrate in HT-PTC is abundant in regulatory T cells and characterised by CXCR3-CXCL10 signalling activation via interferon-γ and NF-κB pathways. Single-cell RNA sequencing has identified a population of HT-specific immune cells/stromal cells (HASCs) that engage through the MIF-CD74-CXCR4 axis, creating a unique TSH-suppressive tumour microenvironment [46,47].

Another issue is the role of pro-inflammatory cytokines. In the TIME coexisting with HT, cytokines secreted by tumour cells, stroma cells, or immune cells play a key role in regulating tumour growth, invasion, and metastasis. Shi et al. found that nine cytokines, including interleukin-1alpha (IL-1α), interleukin-1beta (IL-1β), interleukin-12p70 (IL-12p70), interleukin-8 (IL-8), interferon-induced protein-10 (IP-10), monocyte chemoattractant protein-1 (MCP-1), macrophage inflammatory protein-1alpha (MIP-1α), macrophage inflammatory protein-1beta (MIP-1β), and soluble E-selectin (sE-Selectin) showed significantly higher PTC-HT positive expression in paraneoplastic tissues than HTPTC [48]. In addition, these authors showed that the paraneoplastic tissues of HTPTC patients produced more interferon-alpha (IFN-α) and interferon-gamma (IFN-γ) than tumour tissues. They concluded that HT affects cytokine profiles in PTC patients by stimulating the secretion of Th1-type cytokines and chemokines. These data suggest that CLT in HT promotes an anti-tumour environment that may limit PTC progression.

These studies confirm that many different mechanisms influence the course of TC in HT and provide an explanation as to why, despite the increased risk of PTC in HT, the prognosis of PTC-HT is better.

In our study, we analysed the relationship between histopathologically confirmed CLT and TC. However, the results and subsequent conclusions should be treated with caution, as we did not have data on TSH levels or TPOAb and TGAb, which are necessary for HT confirmation. This was because patients were referred owing to suspected or confirmed malignancy, hyperthyroidism in the course of GBD or TNG, and compressive symptoms. Thus, they were not referred because of HT. Many studies have confirmed the association between TSH levels and the incidence of thyroid cancer [3,9,10,11,12,49,50]. The incidence of PTC increases with increasing TSH [50,51,52] and a meta-analysis including 30 studies by Wang et al. found that increased TC risk is associated with high TSH exposure (OR = 1.28); furthermore, for every 1 mU/L increase in TSH, TC risk increases by 16% [53]. Shahrokh et al. found a statistically significant increase in TSH levels after progression from a benign nodular thyroid disease (BTND) group to a papillary thyroid microcarcinoma (PTMC) group and then to a larger thyroid cancer group. TSH levels were significantly higher in patients with PTC, and an association between high TSH levels and LNM was demonstrated [54]. A large cohort study involving 164,596 patients demonstrated that low TSH levels and elevated FT4 levels were associated with an increased risk of thyroid cancer [55]. Their finding indicates that low levels of TSH and high levels of FT4, even within the normal range, were associated with an increased risk of incident thyroid cancer. Studies on the association between antithyroid antibodies and thyroid cancer have also been undertaken [28,56,57,58,59]. It has been reported that the presence of TPOAb is correlated with an increased incidence of DTC [56]. However, another study indicated that in HT patients, TPOAb acts as a protective factor, whereas TgAb appears to be a risk factor that promotes PTC progression [58]. In another study, high levels of TgAb IgG4 were shown to be a risk factor for the development of PTC, which is related to differences in epitopes [60]. The absence of these data in our study, i.e., TSH or TPOAb and TGAb, is a limitation, so the results should be treated with caution. Furthermore, the group of patients in our study was referred for surgery due to the presence of a focal lesion with a suspicious FNAB result; hence, it was, in part, a pre-selected group. Nevertheless, histopathological examination confirmed the presence of concomitant CLT in addition to the focal lesion, and our study is thus concerned with the relationship between CLT and TC.

In our study, we found that malignant tumours were significantly more common in CLT-positive patients (60.39%) than in CLT-negative patients (34.12%) (*p* < 0.001). The most common type of TC was PTC, which is consistent with data in the literature [3,9,10,11,12,37,38]. When analysing the Bethesda categories of tumours referred for surgery, in the CLT-positive group, Bethesda categories VI (17.39%/8.84%), V (26.57%/11.05%) and IV (14.49%/11.27%) were more frequently found when compared with the CLT-negative group, while categories I-III were less common. In turn, when analysing the Bethesda results in the patients with histopathologically confirmed TC, in the CLT-positive group, there were more categories V (42.40%/28.08%) and VI (28.80%/24.41%), when compared with the CLT-negative group, while categories I-IV were less common. Multivariate analysis confirmed that, after considering the Bethesda categories and age, CLT increases the overall risk of TC (OR = 1.73; 95% CI 1.15–2.29) and PTC (OR = 2.12; 95% CI 1.45–3.11). True-positive FNAB for PTC was more common in the CLT-positive group (74.14%) than in the CLT-negative group (60.77%), which was statistically significant (*p* = 0.009). A similar relationship was found in another study [10]. Differences were also observed in the weight of the removed thyroid gland, which was lower in the Malignancy CLT-positive group compared to the CLT-negative group (*p* < 0.001), which is consistent with the results of another study [10].

In a univariate analysis, compared with the Malignancy CLT-negative group, the Malignancy CLT-positive patients were younger by an average of 7 years (*p* < 0.001), there were more women (*p* < 0.001), the cancer focus was smaller (*p* = 0.013), and microcarcinoma was more common (*p* = 0.021). Similarly, other studies have found younger ages [4,24,25], more female patients [4,24,38], smaller tumour sizes [24,25,45], and more frequent microcarcinoma [29,37,38].

However, in our study, no statistically significant differences were found between the CLT-positive and CLT-negative groups in terms of multifocality, which has also been demonstrated in other studies [23,26,36], although some reports have indicated such an association [4,9]. Dong et al. analysed markers of multifocality in CLT, finding that elevated cytokeratin-19 expression and the BRAF mutations (B-Raf proto-oncogene serine/threonine kinase (BRAF) mutations) indicated multifocal PTC in HT, suggesting the need for total bilateral thyroidectomy in such cases [27]. Furthermore, high TPOAb levels > 1300 IU/mL indicate multifocal PTC in patients with HT, which may be helpful in deciding on total thyroidectomy [28].

The topic of LNM requires separate discussion. In our study, in a univariate analysis, we did not find statistically significant differences across CLT groups of TC patients in terms of LNM (*p* = 0.520) or the number of metastatic nodes (*p* = 0.859). However, although the number of CLNMs in the CLT-positive/CLT-negative group was comparable (12.74%/16.00%), the number of LLNMs in the CLT-positive group was lower than in the CLT-negative group (1.60%/4.97%), which was at the borderline of statistical significance. However, multivariate analysis did not confirm the influence of CLT on the presence of LNM (adjusted OR = 1.09; 95% CI 0.62–1.92) and their location. Several studies have analysed this issue, indicating fewer LNMs in CLT-positive patients [24], but some studies report contrary data [10]. In a study by Min et al., a multivariate analysis showed that four variables, including high serum TgAb levels (>1150 IU/mL), a lower tumour location, and irregular central lymph node (CLN) margins and micro-calcifications in the CLN were significantly associated with CLN metastasis in PTC patients with HT [16]. Conversely, Sun et al. showed that HT was an independent protective factor against tumour invasion and CLNM (OR ≈0.42) [45]. Sus et al. found that HT not only promotes the longitudinal growth of nodules and PTC development, but also reduces the risk of invasion and CLNM [61].

Our analysis also showed that other factors may influence the presence of LNM. Patients with multifocality had a 25% higher probability of developing LNM than the group without multifocality, but the result was not statistically significant OR = 1.25 (95% CI 0.74–2.13). Younger patients (age ≤ 50 years) had a 56% higher risk of metastasis after adjusting for additional factors, but this association was not statistically significant (adjusted OR = 1.56; 95% CI 0.95–2.56). Both before and after adjusting for additional factors, the following had a significant impact on the occurrence of metastases: gender: (adjusted OR = 0.45 (95% CI 0.26–0.77), i.e., women had a 55% lower chance of developing LNM compared to men) and the presence of microcarcinoma: adjusted OR = 0.26; 95% CI 0.16–0.43), i.e., patients with microcarcinoma had a 74% lower chance of developing LNM compared to the group without microcarcinoma.

Assessing the LNM locations (central—CLNM or lateral—LLNM) in the multivariate analysis demonstrated that CLT had no influence. However, other parameters did have an impact, i.e., the presence of microcarcinoma consistently and significantly reduced the likelihood of metastasis in both central and lateral locations (by 64% and 97%, respectively). Younger patients were 1.75 times more likely to develop CLNM, but not LLNM. Female patients were significantly less likely to develop LLNM: women were 77% less likely to develop LLNM, but not CLNM (borderline significance, *p* = 0.08).

Regarding other TC types, compared with the CLT-negative group, in the CLT-positive group, there were fewer FTCs (3.20%/9.29%) and MTCs (0.80%/5.18%), a comparable number of OTCs (2.40%/2.59%), more lymphomas (0.80%/0.43%), and no ATCs. There were only a few of these tumours in our study, which may have influenced the results. Similar results were reported in another study [11].

Finally, we should note the factors that limited our study. These included a lack of TSH, TPOAb, and TGAb values in all patients and a lack of EUTIRADS in most patients, partly because patients were referred for surgery from different medical clinics. Having complete data would undoubtedly increase the substantive value of our study. However, it should be emphasised that the strength of our study lies in the large size of the study group and the very thorough analysis of the HP test confirming or ruling out the presence of CLT. The study has limitations in determining CLT as a genuine risk factor for thyroid cancer, as CLT was diagnosed histologically only after surgery, restricting conclusions about causality or temporal association. Since the analysis includes only patients selected for surgery, CLT cannot be considered a preoperative predictor or a clinical risk stratification tool. A more robust approach, such as a prospective cohort study, would be needed to accurately assess the relationship between preoperative thyroiditis and thyroid cancer risk.

In summary, the preponderance of evidence indicates that HT co-occurrence in PTC patients is associated with a clinically and biologically more favourable disease course. Multiple studies show that PTC with HT is marked by lower tumour stages, fewer nodal metastases, and significantly better survival outcomes. The relative paucity of high-risk molecular alterations (e.g., BRAF^V600E) and the presence of a distinct immunoregulatory microenvironment in HT-PTC underscore the potential protective influence of thyroid autoimmunity. Clinically, these findings support considering HT a positive prognostic factor in risk stratification models rather than as an adverse feature [34,61]. Undoubtedly, further studies on the HT-PTC microenvironment are needed. The discovery of HT-specific stem/progenitor cell (HASC) subtypes is promising. These molecular insights may identify new therapeutic targets and elucidate how autoimmunity shapes tumour behaviour, thereby guiding future clinical trials and treatment [47]. Recognising HT in patients with PTC may refine clinical risk assessment and justify more conservative management. The immunological context plays a key role in thyroid carcinogenesis. It is, therefore, necessary to integrate immunological parameters (e.g., HT status, antibody titres) with traditional clinicopathological factors. Elucidating the interaction between autoimmunity and tumour biology may lead to personalised therapeutic strategies for differentiated thyroid cancer.

## 5. Conclusions

Our findings highlight the importance of awareness regarding the coexistence of CLT and thyroid cancer. Given that CLT is diagnosed histopathologically postoperatively, its role as a preoperative predictor remains uncertain. To optimize surgical decision-making, we recommend assessing thyroid antibodies in cases of elevated TSH before surgery, as they serve as biochemical indicators of thyroiditis. In patients with both positive antibody results and suspected malignant tumours, total thyroidectomy may be a reasonable consideration. However, to draw more definitive conclusions, further investigation through a larger, prospectively collected cohort study is warranted.

## Figures and Tables

**Table 1:** The differences in clinicopathological parameters between patients with and without CLT (n = 1564).

Parameters	CLT (−)	CLT (+)	Total	*p*-Value
	N = 1357 (86.76%)	N = 207 (13.23%)	N = 1564	
Age (years) ^£^				
Mean ± SD	53.1 ± 14.6	47.6 ± 15.0	1564	<0.001
Median (Q1–Q3)	54	46		
	(41.0–65.0)	(36.0–59.0)		
N	1357	207		
Gender n (%) ^§^				
Female	1094 (80.62%)	194 (93.72%)	1288	<0.001
Male	263 (19.38%)	13 (6.28%)	276	
Bethesda category ^§^				
I	51 (3.76%)	3 (1.45%)	54	<0.001
II	551 (40.60%)	51 (24.64%)	602	
III	224 (16.51%)	29 (14.01%)	253	
IV	153 (11.27%)	30 (14.49%)	183	
V	150 (11.05%)	55 (26.57%)	205	
VI	120 (8.84%)	36 (17.39%)	156	
Not performed	108 (7.96%)	3 (1.45%)	111	
Histology ^§^				
Malignancy	463 (34.12%)	125 (60.39%)	588	<0.001
Benign thyroid disease	894 (65.88%)	82 (39.61%)	976	
Thyroid surgery ^§^				
Total thyroidectomy	1160 (85.48%)	195 (94.20%)	1355	
Unilateral lobectomy	182 (13.41%)	12 (5.80%)	194	
Other surgery	15 (1.11%)	0 (0.00%)	15	
Lymph node surgery ^§^				
UCLND	202 (14.91%)	33 (15.94%)	235	
BCLND	442 (32.62%)	112 (54.11%	554	
BLLND	3 (0.22%)	1 (0.48%	4	
CLND and one-sided LLND	49 (3.62%)	6 (2.90%)	55	
BCLND and BLLND	3 (0.22%)	2 (0.97%)	5	
One-sided LLND	13 (0.96%)	0 (0.00%)	13	
Extirpation of lymph nodes	1 (0.07%)	1 (0.48%)	2	
None	642 (47.38%)	52 (25.12%)	694	

Legend: UCLND, unilateral central lymph node dissection; BCLND, bilateral central lymph node dissection; BLLND, bilateral lateral lymph node dissection; CLND and one-sided LLND; central lymph node dissection and one-sided lat. Lymph node dissection; BCLND and BLLND, bilateral central lymph node dissection AND bilateral lateral lymph node dissection; one-sided LLND, one-sided lateral lymph node dissection. ^£^—Mann–Whitney U test. ^§^—Pearson’s chi-square test; *p*-values < 0.05 were considered statistically significant.

**Table 2:** Differences in clinicopathological parameters between patients with and without CLT and malignancy.

Parameters	Malignancy (+) CLT (−)	Malignancy (+) CLT (+)	Total	*p*-Value
	N = 463 (78.75%)	N = 125 (21.25%)	N = 588	
Age (years) ^£^				
Mean ± SD	49.8 ± 15.7	42.9 ± 14.0	588	<0.001
Median	48	41		
(Q1–Q3)	(38.0–62.0)	(32.0–51.0)		
Gender—n (%) ^§^				
Female	364 (78.62)	116 (92.80)	480	<0.001
Male	99 (21.38%)	9 (7.2)	108	
Diameter of the largest foci (mm) ^£^				
mean ± SD	13.3 ± 12.6	9.7 ± 7.7		0.013
Median (Q1–Q3)	9 (6.0–16.0)	8 (5.0–11.0)		
N	443	117	560	
Missing	20	8	28	
Bethesda category—n (%) ^§^				
I	14 (3.02)	1 (0.80)	15	0.019
II	80 (17.28)	12 (9.60)	92	
III	67 (14.47)	14 (11.20)	81	
IV	41 (8.86)	9 (7.2)	50	
V	130 (28.08)	53 (42.40)	183	
VI	113 (24.41)	36 (28.80)	149	
Not performed	18 (3.89)	0 (0.0)	18	
Type of Malignancy—n (%) ^§^				
Papillary Thyroid Cancer (PTC)	379 (81.86)	116 (92.80)	495	
Follicular Thyroid Cancer (FTC)	43 (9.29)	4 (3.20)	47	
Oncotytic Thyroid Cancer (OTC)	12 (2.59)	3 (2.40)	15	
Medullary Thyroid Cancer (MTC)	24 (5.18)	1 (0.80)	25	
Anaplastic Thyroid Cancer (ATC)	3 (0.65)	0 (0.00)	3	
Lymphoma	2 (0.43)	1 (0.80)	3	
Type of Malignancy—n (%) ^§^				
PTC	379 (81.86)	116 (92.80)	495	0.003
Other types	84 (18.14)	9 (7.20)	93	
Subtype of PTC—n (%) ^§^				
Encapsulated variant of PTC	4 (1.06)	2 (1.72)	6	0.176
Follicular variant of PTC	98 (25.99)	20 (17.24)	118	
Classic PTC	247 (65.52)	81 (69.83)	328	
Other unusual variants of PTC	28 (7.43)	13 (11.21)	41	
Positive FNAB (V i VI) for PTC—n (%) ^§^				
No, False Positive	142 (39.23)	30 (25.86)	172	0.009
Yes, True Positive	220 (60.77)	86 (74.14)	306	
Microcarcinoma—n (%) ^§^				
Yes (<10 mm)	231 (49.89)	75 (60.00)	306	0.021
No (≥10 mm)	212 (45.79)	42 (33.60)	254	
Missing	20 (4.32)	8 (6.4)	28	
Multifocality—n (%) ^§^				
Yes (≥2 foci)	97 (20.95)	32 (25.60)	129	0.248
No (1 fucus)	358 (77.32)	90 (72.00)	448	
Missing	8 (1.73)	3 (2.4)	11	
NLNM—n (%) ^§^				
Yes	82 (21.75)	22 (18.97)	104	0.520
No	295 (78.25)	94 (81.03)	389	
pNx + missing	86 (18.57)	9 (7.20)	95	
CLNM/LLNM—n (%) ^§^				
Central LNM (pN1a)	59 (12.74)	20 (16.00)	79	0.065
Lateral LNM (pN1b)	23 (4.97)	2 (1.60)	25	
pN0	295 (63.71)	94 (75.20)	389	
pNx + missing	86 (18.57)	9 (7.20)	95	
Number of metastatic LN ^£^				
Mean ± SD	0.84 ± 2.84	0.76 ± 3.45		0.859
N	455	120	575	
Missing	8	5		
Number of removed LN ^£^				
Mean ± SD	4.75 ± 6.84	5.39 ± 6.17		0.002
N	456	120	576	
Missing	7	5	12	
Total number of metastatic lymph nodes/Total number of lymph nodes on histology ^£^				
Mean ± SD	0.11 ± 0.24	0.08 ± 0.20		0.445
N	389	116	505	
Missing	74	9	83	
T—TNM classification—n (%) ^§^				
I (pT1a + pT1b)	352 (80.55)	106 (87.60)	458	0.155
II (pT2)	63 (14.42)	14 (11.57)	77	
III (pT3a + pT3b)	19 (4.35)	1 (0.83)	20	
IV (pT4a + pT4b)	3 (0.69)	0 (0.00)	3	
pTx	26 (5.62)	4 (3.20)	30	
Total excised thyroid gland weight (gram) ^£^				
Mean ± SD	37.61 ± 91.07	22.48 ± 21.82		<0.001
N	433	118	551	
Missing	30	7	37	

Legend: NLNM, neck lymph node metastasis; CLNM/LLNM, central/lateral neck lymph node metastasis. ^£^—Mann–Whitney U test. ^§^—Pearson’s chi-square test; *p*-values < 0.05 were considered statistically significant.

**Table 3:** Multivariate analysis—assessment of TC probability in the cases of coexisting CLT.

	Crude			Adjusted		
Predictors	β	*p*-Value	OR (95% CI)	β	*p*-Value	OR (95% CI)
Gender (F vs. M)	−0.08	0.56	0.92 (0.71–1.21)	Not included		
CLT (Yes vs. No)	1.08	<0.01	2.94 (2.18–3.97)	0.58	0.01	1.73 (1.15–2.29)
Bethesda (III vs. I or II)	0.88	<0.01	2.42 (1.73–3.38)	0.87	<0.01	2.35 (1.68–3.31)
Bethesda (IV vs. I or II)	0.66	<0.01	1.93 (1.31–2.84)	0.62	<0.01	1.86 (1.25–2.75)
Bethesda (V vs. I or II)	3.75	<0.01	42.68 (26.19–69.55)	3.60	<0.01	36.58 (22.34 –59.89)
Age ≤ 50 (Yes vs. No)	0.95	<0.01	2.51 (2.04–3.10)	0.60	<0.01	1.82 (1.37–2.41)

Hosmer–Lemeshow = 1.13, *p* = 0.88; AUC = 0.78, SE = 0.02; R^2^ McFadenn = 0.248.

**Table 4:** Multivariate logistic regression—assessment of the impact of CLT on the presence of LNM in the malignancy group (n = 588).

	Crude			Adjusted		
Predictors	β	*p*-Value	OR	β	*p*-Value	OR (95% CI)
Gender (F vs. M)	−0.99	<0.01	0.37 (0.22–0.61)	−0.80	<0.01	0.45 (0.26–0.77)
CLT (Yes vs. No)	−0.17	0.52	0.84 (0.50–1.42)	0.08	0.78	1.09 (0.62–1.92)
Microcarcinoma (Yes vs. No)	−1.38	<0.01	0.25 (0.16–0.40)	−1.34	<0.01	0.26 (0.16–0.43)
Multifocality (Yes vs. No)	0.21	0.40	1.24 (0.76–2.03)	0.23	0.40	1.25 (0.74–2.13)
Age ≤ 50 (Yes vs. No)	0.42	0.08	1.52 (0.96–2.42)	0.44	0.08	1.56 (0.95–2.56)

Hosmer–Lemeshow = 4.57, *p* = 0.47; AUC = 0.72, SE = 0.03; R^2^ McFadden = 0.09.

**Table 5:** Multivariate multinomial logistic regression results—effect of CLT on LNM localisation (CLNM vs. LLNM).

N-TNM Types	Predictor	Coeff.	*p*-Value	OR (95% CI)
CLNM (pN1a) vs. pNo				
	CLT (Yes vs. No)	0.19	0.52	1.21 (0.67–2.19)
	Age ≤ 50	0.56	0.05	1.75 (1.01–3.03)
	Gender (F vs. M)	−0.56	0.08	0.57 (0.31–1.06)
	Multifocality (Yes vs. No)	0.23	0.43	1.26 (0.71–2.21)
	Microcarcinoma (Yes vs. No)	−1.03	<0.001	0.36 (0.21–0.59)
LLNM (pN1b) vs. pNo				
	CLT (Yes vs. No)	−0.65	0.40	0.52 (0.11–2.41)
	Age ≤ 50	0.06	0.90	1.06 (0.43–2.61)
	Gender (F vs. M)	−1.45	0.00	0.23 (0.10–0.58)
	Multifocality (Yes vs. No)	0.22	0.67	1.25 (0.45–3.49)
	Microcarcinoma (Yes vs. No)	−3.45	<0.001	0.03 (0.00–0.24)

LR χ^2^ = 66.8, *p* < 0.001; AIC = 567.7; McFadden’s R^2^ = 0.11.

**Table 6:** Multivariate analysis—assessment of PTC probability in cases of CLT coexistence.

	Crude			Adjusted		
Predictors	β	*p*-Value	OR (95% CI)	β	*p*-Value	OR (95% CI)
Gender (F vs. M)	0.24	0.11	1.27 (0.95–1.69)	not included		
CLT (Yes vs. No)	1.21	<0.01	3.34 (2.45–4.54)	0.75	0.01	2.11 (1.37–3.26)
Bethesda (III vs. I or II)	0.78	<0.01	2.18 (1.50–3.17)	0.75	<0.01	2.12 (1.45–3.11)
Bethesda (IV vs. I or II)	0.34	0,15	1.41 (0.89–2.24)	0.26	0.29	1.29 (0.81–2,08)
Bethesda (V vs. I or II)	3.92	<0.01	50.50 (30.65–83.18)	3.75	<0.01	42.64 (25.72 –70.68)
Age ≤ 50 (Yes vs. No)	1.06	<0.01	2.89 (2.31–3.62)	0.74	<0.01	2.10 (1.53–2.87)

Hosmer–Lemeshow = 1.78, *p* = 0.78; AUC = 0.81, SE = 0.02; R^2^ McFadden = 0.277.

## Data Availability

The original contributions presented in the study are included in the article. Further inquiries can be directed to the corresponding authors.
